# Gut phageome: challenges in research and impact on human microbiota

**DOI:** 10.3389/fmicb.2024.1379382

**Published:** 2024-03-22

**Authors:** Xiao Yu, Li Cheng, Xin Yi, Bing Li, Xueqin Li, Xiang Liu, Zhihong Liu, Xiaomei Kong

**Affiliations:** ^1^NHC Key Laboratory of Pneumoconiosis, Shanxi Key Laboratory of Respiratory Diseases, Department of Pulmonary and Critical Care Medicine, The First Hospital of Shanxi Medical University, Taiyuan, China; ^2^Department of Clinical Laboratory and Pathology, Hospital of Shanxi People’s Armed Police, Taiyuan, China; ^3^Academy of Medical Sciences, Shanxi Medical University, Taiyuan, China; ^4^Department of Pulmonary and Critical Care Medicine, The General Hospital of Jincheng Coal Industry Group, Jincheng, China

**Keywords:** gut microbiome, gut phages, interactions, composition, phage identification, lysogenic phages

## Abstract

The human gut microbiome plays a critical role in maintaining our health. Fluctuations in the diversity and structure of the gut microbiota have been implicated in the pathogenesis of several metabolic and inflammatory conditions. Dietary patterns, medication, smoking, alcohol consumption, and physical activity can all influence the abundance of different types of microbiota in the gut, which in turn can affect the health of individuals. Intestinal phages are an essential component of the gut microbiome, but most studies predominantly focus on the structure and dynamics of gut bacteria while neglecting the role of phages in shaping the gut microbiome. As bacteria-killing viruses, the distribution of bacteriophages in the intestine, their role in influencing the intestinal microbiota, and their mechanisms of action remain elusive. Herein, we present an overview of the current knowledge of gut phages, their lifestyles, identification, and potential impact on the gut microbiota.

## 1 Introduction

The human gut contains a diverse range of microorganisms-bacteria, archaea, eukarya, and viruses, that play important roles in various life activities to maintain the health of the host (Zhang et al., 2023). Numerous factors influence the homeostasis of gut microbes, including the host’s own age, dietary habits, physical activity, disease state and medication use (Pargin et al., 2023). Presently, microbiome studies have almost exclusively focused on bacteria instead of other species. The gut virome is the community of all viruses found in the gut, including bacteriophages, eukaryotic viruses, and human-specific viruses (Hannigan et al., 2018). Although the number of viruses is relatively lower in the composition of the gut microbiome, the number of intestinal phages has been reported to be 109 virus-like particles (VLPs) per gram of human feces, one to two orders of magnitude greater than the number of gut bacteria (Dion et al., 2020). Bacteriophages may be crucial in shaping microbial composition, driving bacterial diversity, and promoting horizontal gene transfer. Nonetheless, the mechanism by which the virome and the microbiome influence each other remains largely unknown. Thus, this article aims to review the current research progress on the interrelationship between phages and the intestinal microbiota.

## 2 Discovery, classification, and characteristics of the phage

**Figure 1:**
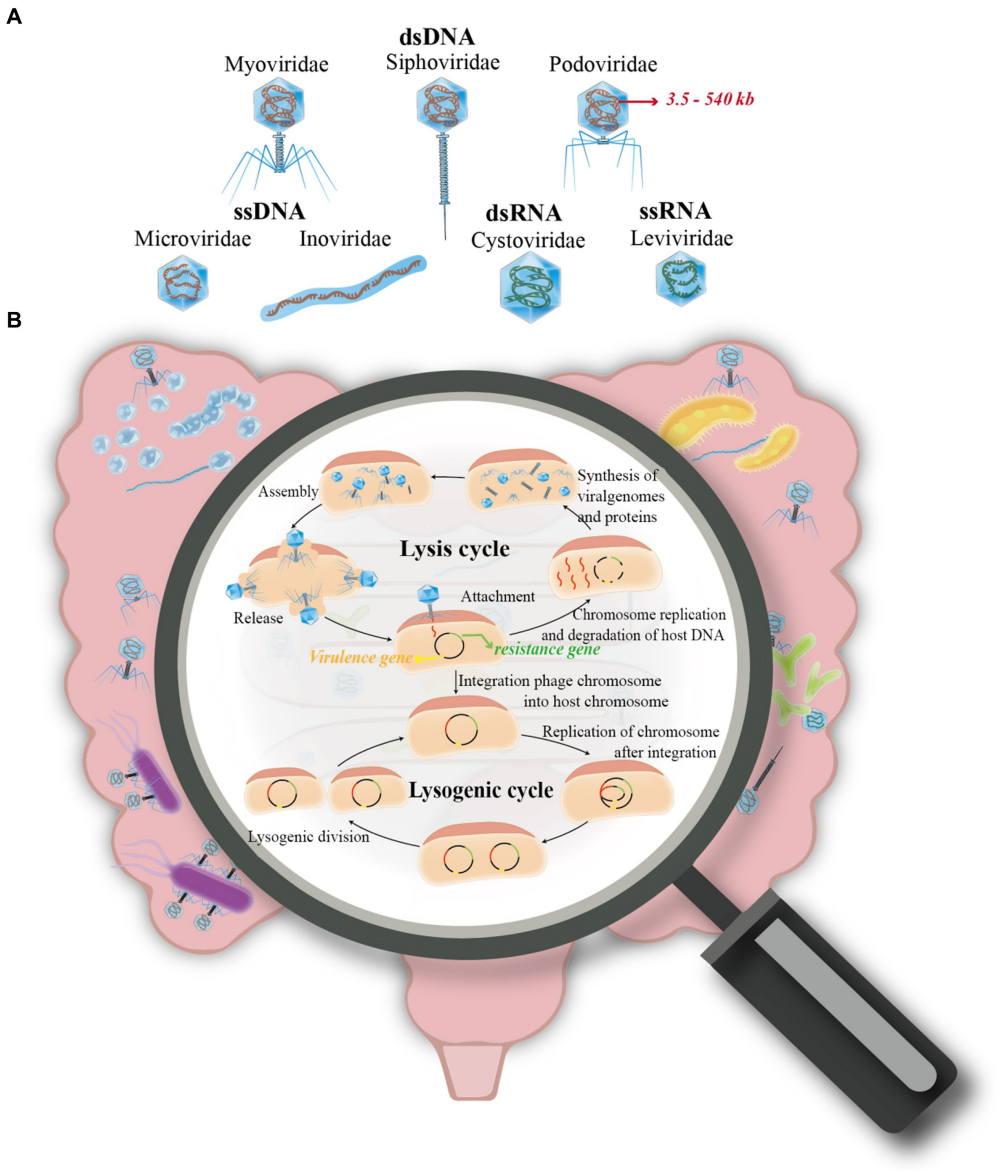
Overview of phage life cycles and classification. **(A)** This section of the figure classifies phages into four primary groups based on their nucleic acid types. These include: (i) Double-stranded DNA (dsDNA) phages, incorporating families such as Siphoviridae, Myoviridae, and Podoviridae. (ii) Single-stranded DNA (ssDNA) phages, represented by families like Microviridae and Inoviridae. (iii) Double-stranded RNA (dsRNA) phages, which belong to the Cystoviridae family. (iv) Single-stranded RNA (ssRNA) phages, exemplified by the Leviviridae family. **(B)** The second part of the figure illustrates the two primary life cycles a phage may undertake after infecting a host cell: (i) The Lysis Cycle: Here, the phage’s DNA (or RNA) replicates, transcribes, and expresses its genes within the host cell. This leads to the assembly of new phage progeny, which eventually cause the host cell to lyse (rupture) for release, or exit through extrusion. (ii) The Lysogenic Cycle: Contrary to the lysis cycle, the phage integrates its genetic material into the host’s chromosome. This integration allows the phage to replicate along with the host cell’s DNA during cell division.

In 1915, Twort first discovered the presence of bacteriophage and analyzed the process of killing bacteria in solid medium plates (Duckworth, 1976). Phage infection is typically followed by one of two replication cycles, lytic or lysogenic and are referred to as virulent and mild phages, respectively. The mild phages integrate nucleic acid into the host bacterium’s genome and use its host’s genome to produce a prophage. In this condition, no zygotic phage can be produced, but the phage genes are integrated with the host chromosome, and the phage undergoes DNA replication with the host and is passed on as the host divides (Howard-Varona et al., 2017; Gildea et al., 2022; Avellaneda-Franco et al., 2023). In contrast, the virulent phage undergoes replication independently of the host bacterial DNA during the lytic cycle. This process involves transcription for synthesizing protein shells, followed by assembly to produce numerous progeny phages. Consequently, this leads to the destruction of the infected bacterium, culminating in the lysis of the bacterium and the release of a substantial quantity of phages (Dang et al., 2015; Townsend et al., 2021) (Figure 1B).

According to the nucleic acid type of the phage, it can be classified into double-stranded (ds) DNA (Myoviridae, Siphoviridae and Podoviridae), single-stranded (ss) DNA (Microvirdae, Inoviridae), dsRNA (Cystoviridae), or ssRNA (Leviviridae), with more than 95% being dsDNA phages. Besides, the genome sizes of phages range from ∼3.5 kb to ∼540 kb (Sausset et al., 2020) (Figure 1A). In addition, phages are also classified based on capsid morphology, such as the presence or absence of an envelope or tail. This classification includes three primary types: the tailed phage, which features an ortho-polyhedral head and a complex tail comprising a hollow, needle-like structure with an outer sheath and a base made of a tail filament and tail pin; the tailless phage, characterized by its positive polyhedral shape with an exterior of regularly arranged protein subunits (capsids) encapsulating nucleic acids; and the filamentous phage, distinguished by its linear shape and lack of a distinct head structure, instead having a coiled structure composed of shell grains (Feyereisen et al., 2019).

## 3 Current challenges of phage identification

Historically, research on the phageome within the gut microbiome has been constrained, primarily due to the limitations of the research tools available at that time. These tools included the direct observation and counting of virus-like particles (VLPs) using advanced microscopic techniques such as transmission electron microscopy (TEM), scanning electron microscopy (SEM), and epitaxial fluorescence microscopy (EFM). Additionally, the isolation of individual phages that infected specific host strains was commonly performed through culture methods (Bai et al., 2022). While these microscopic methods have been instrumental in uncovering the diversity of viral morphologies, they have been less effective in accurately quantifying the total number of bacteriophages present in human feces, cecum contents, and colonic mucosa, which is estimated to be approximately 109 to 1,010 VLPs per gram (g-1) (Shkoporov and Hill, 2019).

Ever since the identification of phages, culture-based methods have been employed to screen and quantify phages; however, most gut viruses (mostly phages) are strictly dependent on their hosts and are not amenable to culture with common microbiological techniques (Forster et al., 2019). The development of high-throughput sequencing and metagenomic methods has allowed the reconstitution of gut microbiota composition from single genetic sequences (Wang et al., 2023). Currently, metagenomic sequencing has become the gold standard for studying gut microbiomes. Computational approaches have also been applied to extract species and even subspecies-level information from metagenomic sequences, enabling the characterization of taxonomic-level bacterial compositions (Scholz et al., 2016). Nevertheless, the fragment assembly performance of phages from metagenomic sequencing data is not as robust as that of bacteria, causing the assembly sequences of phages to exist as a large number of short fragments, thereby making identification challenging (Rozov et al., 2017). There are two main strategies to address this issue. The first strategy is to extract the whole genome from the sample-enriched virome, which can be achieved through the purification of viral particles and elimination of all cells and free-floating nucleic acids by filtration, centrifugation, and enzymatic reactions (Conceição-Neto et al., 2015).

**Figure 2:**
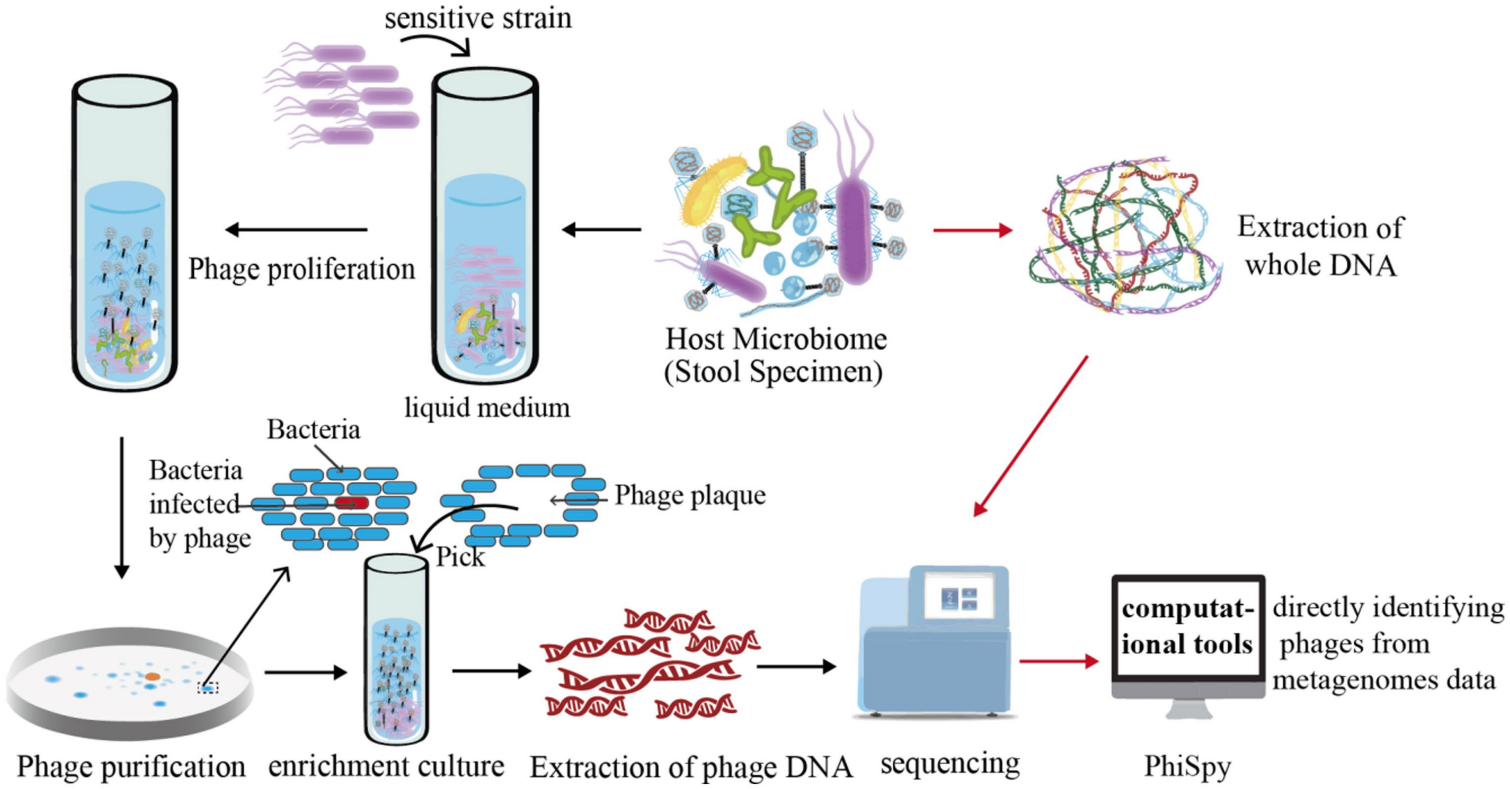
Illustration of phage research methodologies. This figure delineates the methodologies employed in isolating and studying phages. Initially, intestinal samples are extracted and amalgamated with bacterially sensitive strains. This combined specimen is then cultured in a liquid medium, a step crucial for fostering the growth and release of a large quantity of phages. For visualization, the figure shows how a meticulously measured mix of phage and sensitive bacteria is incorporated into soft agar, then layered onto a solid agar medium for incubation. The interaction between the phage and bacteria during this phase leads to the lysis of bacterial colonies, forming distinctive clear zones known as “phage plaques.” These plaques are pivotal for phage purification as, ideally, a single phage particle can produce one plaque. For advanced analysis, the figure further explains that phages are cultivated in significant numbers to extract phage DNA. This DNA undergoes high-throughput sequencing, followed by comprehensive bioinformatics analysis for deeper insights. Additionally, computational tools like PhiSpy, a marker gene-dependent phage detection algorithm, are depicted as instrumental in identifying phages directly within metagenomic datasets.

Initially, host bacteria are isolated and cultured from biological samples, which are then mixed with sensitive strains and cultured in liquid media. Phages are proliferated and released in significant quantities. Subsequent centrifugation of the culture isolates the supernatant, which is then mixed with sensitive bacteria in soft agar and evenly spread over an agar medium. In the presence of phages, the bacteria on the culture medium’s surface undergo lysis due to phage activity, resulting in transparent, sterile circular voids known as phage plaques. A single phage plaque is selected, diluted, and incubated on a double-layer plate. Following the growth of the phage plaque, another single plaque is picked and the dilution and culturing process repeated 5–6 times for phage purification. The uniformity in the morphology of phage plaques grown on the plate serves as the criterion for phage purification. To obtain a sufficient quantity of phage particles or extract ample phage DNA, phages are enriched using a liquid medium (Yu et al., 2015; Luong et al., 2020) (Figure 2).

## 4 Bioinformatic tools in phageome analysis

The enriched samples lack host information, but enrichment techniques require a certain level of expertise and experience. The second strategy, computational tools for directly identifying phages from metagenomes data, are expected to be developed in the near future (Fang et al., 2019) (Figure 2). Based on the methods used to identify phages, these software can be divided into three distinct categories. The first one relies on known marker phage genes. For instance, PhiSpy, a phage detection algorithm, was developed based on several features, including phage protein length, transcribed strand directionality, CG skewness, abundance of phage-specific word lengths, phage insertion sites, and phage protein similarity (Akhter et al., 2012). The second method leverages the spacer sequences inherent to the bacterial CRISPR/Cas system. Paola et al. have employed these CRISPR/Cas-derived sequences as a means to identify bacteriophages effectively (Soto-Perez et al., 2019). Lastly, the third method, which employs deep learning algorithms, is exemplified by tools such as “VirFinder” and “MARVEL.” “VirFinder” utilizes k-mer based analysis for identifying viral sequences in assembled metagenomic data (Ren et al., 2017) while “MARVEL” predicts bacteriophage sequences within metagenomic bins (Amgarten et al., 2018). These approaches enable the identification of phage sequences from complex metagenomic datasets (Ren et al., 2017; Amgarten et al., 2018).

## 5 Characteristics and composition of the intestinal phageome

About half a century ago, researchers discovered a rich and diverse community of non-pathogenic viruses (primarily phages) colonizing the mammalian gut (Donaldson et al., 2016). Until the last decade, while the bacterial composition of the microbiome has received considerable attention, relatively little was known about the composition and physiological significance of the human gut phage. This knowledge gap was chiefly due to the limited tools and technical means available (Clinton et al., 2022). Gut viromes can only be identified using traditional culture-based methods followed by microscopy techniques such as transmission electron or epifluorescence microscopy. However, these methods are not applicable for prophages. With advancements in high-throughput genome sequencing, we can now explore the unique ecological characteristics of the human gut virome (Zhao et al., 2019).

Metagenomic datasets have indicated that phages exist at levels comparable to their bacterial hosts in the human gut (Looft et al., 2014). The gut contains a diverse bacteriophage community, mostly unique to each individual and largely uncharacterized (Tetz et al., 2019). It is now known that the enterophage group consists mainly of DNA phages, predominantly Caudovirales (dsDNA viruses), followed by Microviridae (ssDNA viruses) (Cao et al., 2022). The most globally distributed intestinal phage identified in the current study is crAssphage, which infects Bacteroides and has been hypothesized to be a stable colonizer in the human gut (Dutilh et al., 2014; Koonin and Yutin, 2020). Considering the limited studies on phages, the existence of a “core phageome” is questionable. However, healthy individuals tend to conserve the same phages over time, accounting for approximately 90% of the gut virome (Minot et al., 2011; Shkoporov et al., 2019).

The abundance of phages within different regions of the gut is significant, varying with dietary influences, the presence of certain bacteria, gut health, and disease states. This abundance is subject to fluctuations, necessitating targeted metagenomic studies to ascertain exact levels. The literature acknowledges phages’ considerable presence and potential impact on the gut ecosystem. The physiological characteristics of the gut result in distinct microbial communities and bacteriophages. For example, the proximal gut presents a microaerophilic environment that fosters bacterial families like Lactobacillaceae and Enterobacteriaceae, while the distal gut, being anaerobic, allows Bacteroidaceae, Prevotellaceae, and Ruminococcaceae to thrive (Donaldson et al., 2016). This environmental gradient likely affects phage distribution and abundance, with empirical evidence from primate and porcine models showing site-specific variations in phage abundance and composition within the gastrointestinal tract (Looft et al., 2014; Zhao et al., 2019; Clinton et al., 2022). Phage populations also vary cross-sectionally through the gut’s mucus layers, which may have further implications for the Immune System (Tetz et al., 2017).

Disease conditions have the potential to alter both the abundance and types of phages present within the gut microbiome. Shifts in the microbiome due to illness can prompt changes in the populations of phages, contingent on the available bacterial hosts for infection. Diseases can reduce the diversity of gut bacteria, potentially leading to a corresponding decrease in phage diversity. Conversely, some conditions, such as diabetes, have been observed to increase populations of specific bacteria like *E. coli*, which in turn may elevate the numbers of associated phages (Tetz et al., 2019). Understanding the dynamic interplay between bacteriophages, their bacterial hosts, and the health of the host organism is complex and currently a subject of intensive study, considering the implications for both phage populations and host health.

Furthermore, phage predation of gut bacteria — especially those with protective roles in human health — can contribute to dysbiosis and disease (Diard et al., 2017). However, phages also play a critical role in controlling populations of invasive bacteria and in maintaining the integrity of the intestinal barrier function (Barr et al., 2013). Conversely, an increase in gut phage populations can directly affect intestinal permeability, potentially leading to the translocation of bacteria and bacterial products into the bloodstream and exacerbating chronic inflammatory responses (Tetz et al., 2017). The precise impact of phages on gut health thus remains a delicate balance, one that requires further elucidation for potential therapeutic applications.

## 6 Interactions between phages and bacteria

Similar to intestinal microbiota, fluctuations in the composition and functionality of gut phageome affect the health of the host. Although data regarding the impact of phages on the gut microbiota ecosystem are growing, there is still a lack of understanding (Zhang et al., 2023). It is essential to determine whether phages merely react to changes in the microbiota composition or if they actively shape the bacterial dynamics within the gut ecosystem. In many environmental ecosystems, dynamics of phages following the “kill the winner” theory, bacteriophages expand on the fastest growing host population; when the host population declines, bacteriophage replication is no longer supported, and the increase in the number of phages stops. Therefore, microbial diversity is maintained, and no particular species can dominate the ecosystem (Thingstad, 2000). However, “kill-the-winner” dynamics were not reported for phage-bacterial interactions in the gut (Reyes et al., 2010). Ultimately, the spatial location and physiological status of the cells determine the interaction between phages and bacteria. For example, *Escherichia coli* are usually nutritionally-deprived and non-replicating in the lumen of the colon, whereas they are metabolically active in the mucus layer of the intestinal mucosa. Indeed, *E. coli* strains in the former location are a poor target for phages (Chibani-Chennoufi et al., 2004).

The vast majority of phages interact in species-level specificity with bacterial strains. The relationship between phages and host bacteria may involve mutual exploitation. Gut phages generally either help their host or kill them, and this equilibrium changes rapidly over time. Meanwhile, the survival of phages also depends on the bacterial host. To further elaborate, phages in the gut can be categorized into lysogenic and lytic types, each playing distinct roles in their interaction with bacterial hosts.

## 7 Benefits of lysogenic phages to host gut bacteria

The majority of phages found in the human gut are prophages, which exhibit a typical “temperate” behavior, and their composition is stable during the host’s life (Kim and Bae, 2018). The genome fragment rearrangements occur in gut microbiota as a consequence of homologous recombination, and prophages serve as the anchor points (Nakagawa et al., 2003). The insertion of phages could disrupt bacterial genes, thereby suppressing gene functions (Lam et al., 2012). Additionally, prophages also encode fitness-enhancing genes that enable host bacteria to expand their environmental niche, giving them an advantage. These fitness-enhancing genes are not crucial for the life cycle and are highly variable from phage to phage. It is well established that phage predation and lysogenic transformation in bacterial populations play a key role in horizontal gene transfer and regulating bacterial abundance. A huge body of evidence shows that phages can impart genetic diversity that can benefit bacterial cells (Leigh, 2019; Wahl et al., 2019; Avellaneda-Franco et al., 2023).

Transduction of genes for toxins and antibiotic resistance by phages is well documented, as in the case of the Shiga toxin and several antibiotic resistance genes (Brüssow et al., 2004; Johnson et al., 2022). Prophages encoding genes can lyse related strains to reduce competition (“kill-the-relatives”) (Fortier and Sekulovic, 2013). Besides, during intestinal phageome evolution, stably conserved genes discovered are those implicated in energy harvestings such as for carbohydrate transport and degradation (Markine-Goriaynoff et al., 2004; Minot et al., 2011). These phages containing energy-harvesting genes could modulate human gut microbiota and metabolism when located in the host genome (Reyes et al., 2010). It has been previously reported that prophage induction promotes Vibrio eel biofilm formation at low cell density in the early stage of infection while facilitating the increase of the number of bacteria in the later stage of infection (Tan et al., 2020). Several genes mediate host bacterial resistance to further phage infection, including altering the lipopolysaccharide (LPS) to prevent phage binding (Krylov et al., 2013) or modifying the O-antigen to block phage superinfection (Perry et al., 2009). Finally, numerous phages have been identified to contain genes that affect bacterial motility in a strain-specific manner (Tsao et al., 2018) and activate or repress the virulence of host bacteria (Gong et al., 2024).

## 8 Impact of lytic phages on gut microbiota

In addition to lysogenicity, phages can directly influence the gut microbiome by depleting target microbes. Some environmental stressors, such as mutagens or inflammation in the gastrointestinal tract, can promote prophage release (Górski et al., 2018). The phage injects viral genetic material into the target bacteria by adsorbing its tail to the cell wall of the target bacteria. The viral genetic material utilizes the host bacteria for DNA replication and protein synthesis, and then generates a progeny phage through assembly. After the progeny phage matures, the peptidoglycan layer of the host bacterial cell wall is degraded in large quantities by the action of phage lytic proteins (endolysins), leading to bacterial lysis and release of the progeny phage (Criel et al., 2021; Emencheta et al., 2023). After infecting a target bacterium, the phage can rapidly generate hundreds of progeny phages, and each progeny phage can infect the surrounding target bacteria and generate hundreds of progeny phages, and so on for several times, which can lead to the death of a large number of target bacteria, thus affecting the intestinal microbiome. Bacteriophages (phages) are often described as specialized predators of their host bacteria, and phage predation is one of the major forces controlling the density and distribution of bacterial populations (Shkoporov et al., 2022). Phage predation directly affects the corresponding susceptible bacteria and even exerts a cascading effect on other non-targeted bacterial species reported in a mouse model. In this study, the authors, using a broad metabolic profile, demonstrated that changes in bacterial composition caused by phage predation can also modulate the gut metabolome (Hsu et al., 2019). These findings have implications for mammalian hosts and may lead to the application of phages for therapeutic purposes. It is worthwhile noting that owing to the unclear bacterial protection mechanism of the gut and the rapid evolution of the anti-phage mechanism in the host, phages can barely fully eradicate a host bacterial species. Thus, fluctuations in gut microbiota composition caused by phages at the species level are expected to be transient (Nanda et al., 2015). This phenomenon indirectly implies that microbial community formation is a more stable system and that phage predation may be beneficial in increasing the stability of bacterial communities. Phages are considered top candidates for the rational adjustment of the gut microbiome due to their function as specific bacterial antagonists. The deletion of gut bacterial strain by phages has been attempted in several ways; however, the efficacy of this biological technology remains inconclusive (Sarker et al., 2016; Gundersen et al., 2023). The ability of phages to regulate the gut microbiome has shown great promise, but findings are limited by current microbial complexity (Reyes et al., 2013). Despite its potential, the effective modulation of the gut microbiome using phages warrants further investigation and experimental evidence.

## 9 Phage dynamics and immune interactions in the gut

Given that the phage-bacteria relationship is a predator–prey relationship, it is often assumed that changes in the intestinal microbiota affect the phage composition. However, Ma et al. discovered that the number of phages in the intestines of diabetic patients was significantly higher than that in the healthy control group, and further analysis uncovered a complex network of relationships between intestinal bacteria and phages. The alterations of the gut phageome cannot be explained simply by co-variation with the altered bacterial hosts (Ma et al., 2018). Previously, phages were regarded as bystanders that only impacted human immunity indirectly via the gut microbiome. However, it is now apparent that phages could interact directly with the host immune system (Popescu et al., 2021). Direct interactions between phages and mammalian cells typically induce host inflammatory and antiviral immune responses. This can occur directly through phage internalization and receptor recognition by mammalian cells. Phages are actively internalized by the host cell and activate conserved virus-detecting receptors, leading to secretion of proinflammatory cytokines and recruitment of adaptive immune programs (Champagne-Jorgensen et al., 2023). The presence of whole phage particles and their components, including genomic DNA or RNA, protein capsids, and residual bacterial products, such as lipopolysaccharide (LPS), can directly stimulate the mammalian immune response (Wahida et al., 2021). Gogokhia et al. found that treatment of germ-free mice with phage resulted in the amplification of immune cells in the gut. Elevated phage levels exacerbate intestinal inflammation in colitis and are mediated by activation of phage-specific and non-specific IFN-γ-mediated immune responses via TLR9 receptors. Phages can directly activate mammalian immunity without the involvement of host bacteria (Gogokhia et al., 2019). In addition, phages can also directly inhibit bacterial growth as a bio-barrier when they adhere to gut mucosa using their outer shell proteins. These phages could assist in eliminating pathogenic bacteria that invade the mucosa (Gliźniewicz et al., 2024).

## 10 Conclusions and future prospects

Phageome can modify the composition and function of the gut microbiota in numerous ways. Lysogenic conversion involving fitness genes such as the antibiotic, virulence, and energy genes is potentially the most determining contribution of prophages to the host bacteria. These fitness genes, gene inactivation caused by phage insertion, and gene rearrangement together affect the abundance of the host bacteria in the intestinal microbiota. Notably, lytic phages act as a selective pressure in the gut microbiome, regulating microbiota structure and metabolism by reducing the number of target species. It is imperative to further delineate the mechanisms governing the prolificacy of bacteriophages that function autonomously from the intestinal microbiota, alongside assessing their unequivocal implications on the immunological defense mechanisms of the host.

During the last decade, advances in metagenomics have shed light on the composition and dynamics of the viral component of the gut microbiota. Indeed, high-throughput sequencing and novel assembly methods have allowed the description and identification of new phages. These methods have notably revealed that the virome composition is highly variable, with only a small fraction of gut phages shared among individuals. This novel classification and uniform criteria of these gut phageome, which is vital for understanding their roles in the gut microbiome, need to be established. Furthermore, the low rate of identification of intestinal phages and the lack of a comprehensive understanding of the factors that contribute to the stability of the intestinal microbiota are two of the greatest obstacles to characterizing the intestinal microbiota. The life cycle of phages makes it difficult to determine their possible roles in shaping the gut microbiota. The first two challenges are gradually being addressed with increased research and advances in sequencing and analysis techniques. However, innovative ideas about the conversion in the lytic or lysogenic infection of bacteria and its impact on the microbiota are currently needed.

## Author contributions

XiaY: Writing – original draft, Writing – review & editing. LC: Writing – review & editing. XinY: Writing – review & editing. BL: Writing – review & editing. XuL: Writing – review & editing. XiL: Writing – review & editing. ZL: Writing – review & editing. XK: Writing – review & editing.
